# Supplementary material for: Identification and Characterisation of pST1023 A Mosaic, Multidrug-Resistant and Mobilisable IncR Plasmid
Source: Microorganisms. 2022 Aug 8;10(8):1592. doi: 10.3390/microorganisms10081592 (PMC9412624; doi:10.3390/microorganisms10081592)
Supplement: Supplementary file 1 [file microorganisms-10-01592-s001.zip › Supplementary Figure S1.pdf]

Figure S1: Locus A

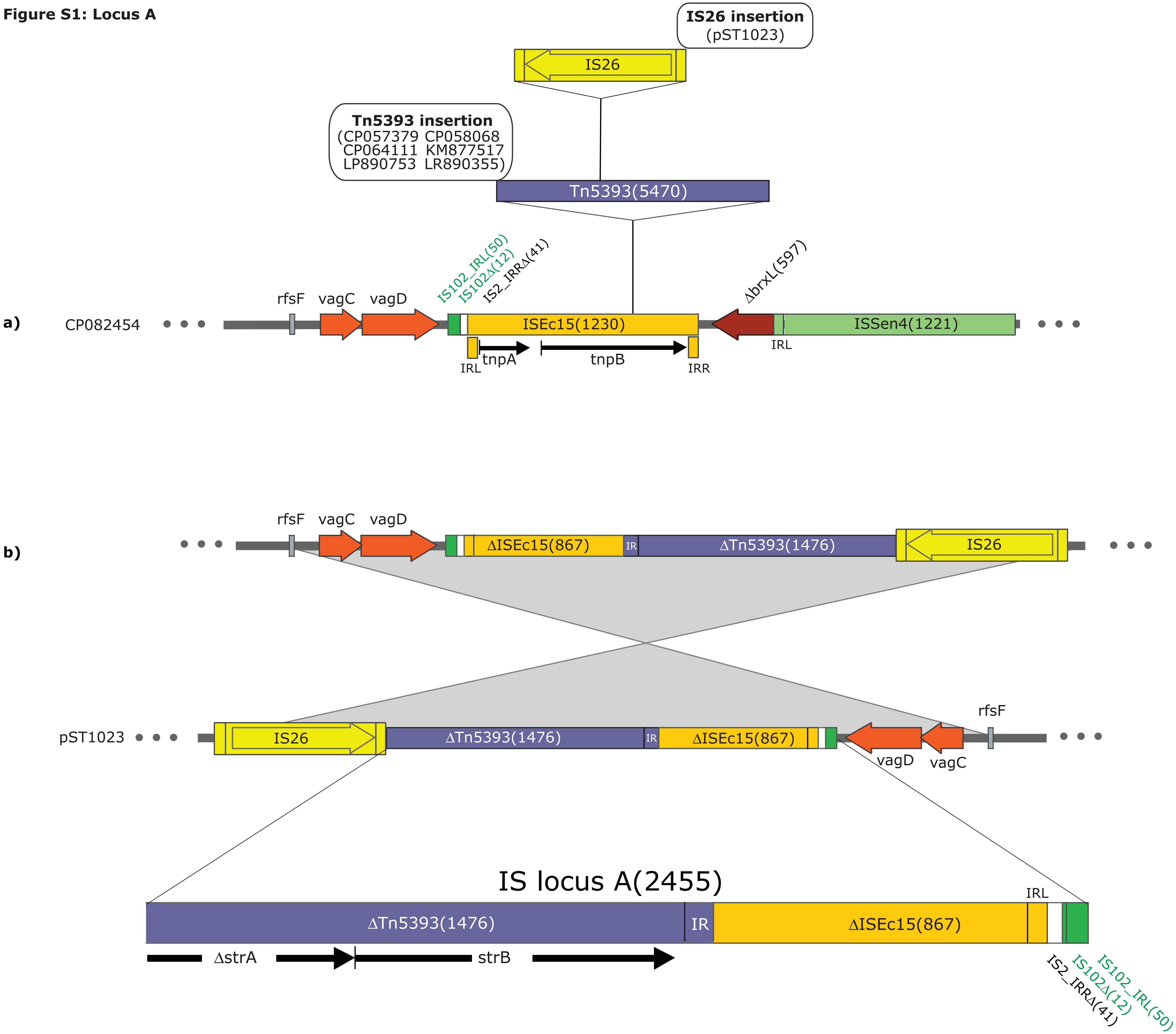

Supplementary Figure S1. Locus A.
IncR plasmids harbouring sequences related to locus A. Arrows indicate the 5′ to 3′ transcription direction. Within square brackets are reported the number of bp. a) plasmid pF18S020 (GenBank Acc. N° CP082454) harbouring an intact IS*Ec15*. IncR plasmids (GenBank Acc. N° CP057379, CP058068, CP064111, KM877517, LR890355 and LR890753) with the IS*Ec15* disrupted by Tn*5393* (same nucleotide position as in pST1023). Disruption of Tn*5393* by IS*26* as found in pST1023. b) Locus A in pST1023.
