# Supplementary material for: Identification and Characterisation of pST1023 A Mosaic, Multidrug-Resistant and Mobilisable IncR Plasmid
Source: Microorganisms. 2022 Aug 8;10(8):1592. doi: 10.3390/microorganisms10081592 (PMC9412624; doi:10.3390/microorganisms10081592)
Supplement: Supplementary file 1 [file microorganisms-10-01592-s001.zip › Supplementary Figure S2.pdf]

Figure S2: Locus B

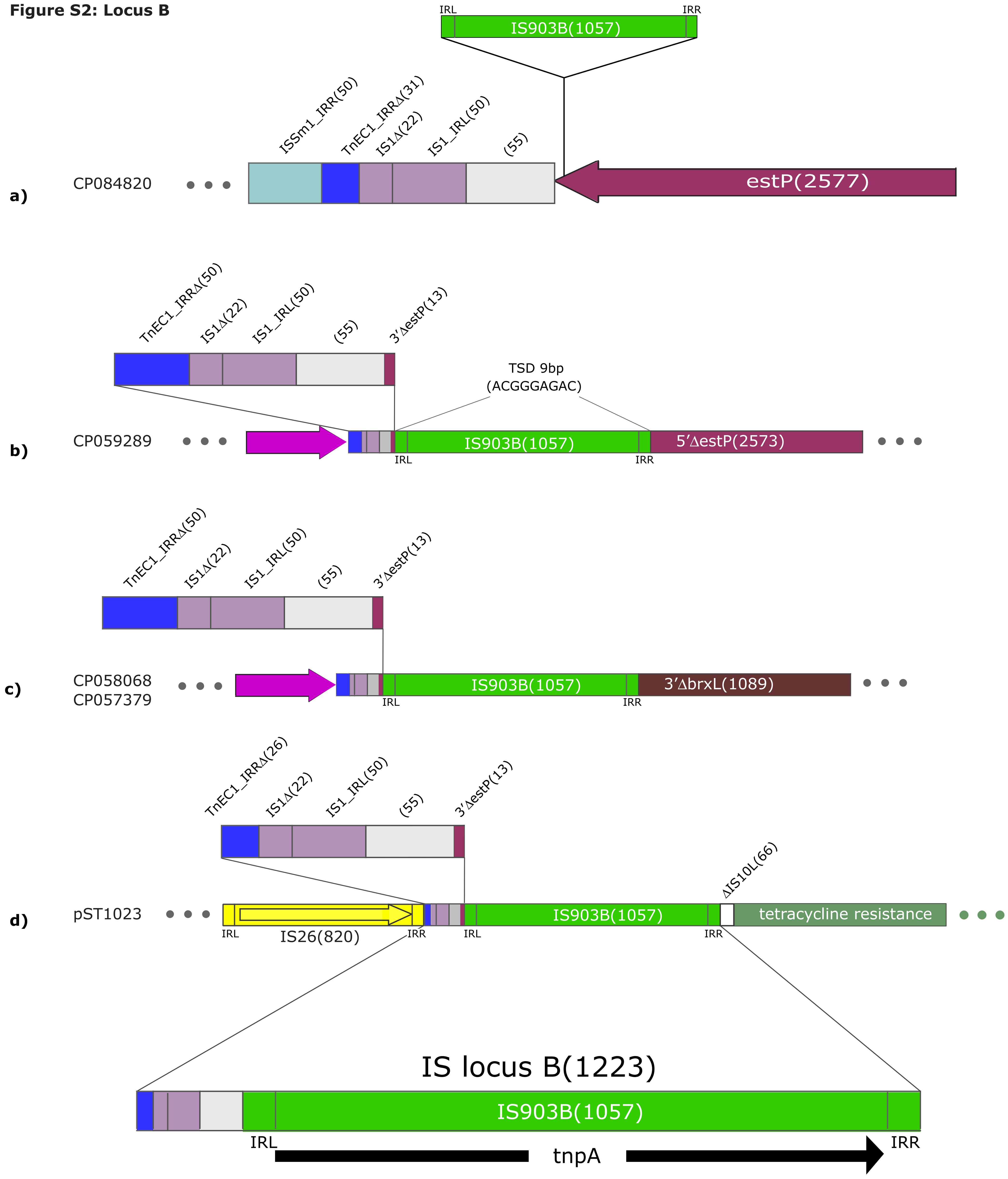

Supplementary Figure S2. Locus B.

Plasmids harbouring sequences related to locus B. Within square brackets are reported the number of bp. a) plasmid pKqq_18A069_2 (GenBank Acc. N° CP084820) carrying a ΔIS*1*-Tn*Ec1* separated from the *estP* gene by a 55bp fragment. b) in plasmid p1506-1 (GenBank Acc. N° CP059289) the insertion of IS*903B* disrupt *estP* gene. The 9bp TDS sequence is reported. The locus B is flanked by *5'ΔestP*.
c) plasmids pRHB02-C19_6 and pRHB28-C14_2 (GenBank Acc. N° CP058068 and CP057379, respectively) harbour the locus B flanked by *3'ΔbrlxL*. d) locus B in pST1023 was flanked by Tn*10* derived sequence.
