# Supplementary material for: Identification and Characterisation of pST1023 A Mosaic, Multidrug-Resistant and Mobilisable IncR Plasmid
Source: Microorganisms. 2022 Aug 8;10(8):1592. doi: 10.3390/microorganisms10081592 (PMC9412624; doi:10.3390/microorganisms10081592)
Supplement: Supplementary file 1 [file microorganisms-10-01592-s001.zip › Supplementary Table S1.pdf]

Table S1: ISFinder blast results

INSERTION SEQUENCES								
**pST1023**			**IS FINDER RESULTS**					
NAME	LENGHT	POSITION	FAMILY	NAME	LENGHT	IDENTITIES	GAPS	
IS*1X2*	768	119,546-120,313	IS1	IS*1X2*	768	748/768 (97%)	0/768 (0%)	
IS*1X3*	769	3788-4556	IS1	IS*1X3*	769	769/769 (100%)	0/769 (0%)	
IS*1N*	766	64,723-65,487	IS1	IS*1N*	765	634/754 (84%)	1/754 (0%)	
IS*930B*$_1$	1057	987-2043	IS5	IS*930B*	1057	1008/1057 (95%)	0/1057 (0%)	
IS*930B*$_2$	1057	83,111-84,167	IS5	IS*930B*	1057	1005/1057 (95%)	0/1057 (0%)	
IS*Kpn74*	1056	115,154-116,209	IS5	IS*Kpn74*	1056	1049/1056 (99%)	0/1056 (0%)	
IS*26*$_1$	820	36,554-37,373	IS6	IS*26*	820	820/820 (100%)	0/820 (0%)	
IS*26*$_3$	820	82,125-82,944	IS6	IS*26*	820	820/820 (100%)	0/820 (0%)	
IS*26*$_5$	820	100,660-101,479	IS6	IS*26*	820	820/820 (100%)	0/820 (0%)	
IS*26*$_6$	820	116,823-117,642	IS6	IS*26*	820	820/820 (100%)	0/820 (0%)	
IS*26*$_{2\text{-V1}}$	820	69,880-70,699	IS6	IS*15D1*	820	820/820 (100%)	0/820 (0%)	
IS*26*$_{4\text{-V1}}$	820	90,643-91,462	IS6	IS*15D1*	820	820/820 (100%)	0/820 (0%)	
